# Erythropoetin receptor expression in the human diabetic retina

**DOI:** 10.1186/1756-0500-2-234

**Published:** 2009-11-25

**Authors:** Shaival S Shah, Stephen H Tsang, Vinit B Mahajan

**Affiliations:** 1 Vitreoretinal Service, Department of Ophthalmology and Visual Sciences, The University of Iowa Hospitals & Clinics, 200 Hawkins Drive, USA; 2 Omics Laboratory, Iowa City, IA, USA; 3 Bernard and Shirlee Brown Glaucoma Laboratory, Department of Pathology and Cell Biology, Department of Ophthalmology, College of Physicians and Surgeons, Columbia University, New York, New York, USA

## Abstract

**Background:**

Recent evidence suggests erythropoietin (*EPO*) and the erythropoietin receptor (*EPOR*) may play a direct role in the pathogenesis of diabetic retinopathy. Better characterization of the *EPO-EPOR *signaling system in the ischemic retina may offer a new therapeutic modality for ischemic ophthalmic diseases. This study was performed to identify *EPOR *mRNA expression in the human diabetic eye.

**Findings:**

*EPOR *antisense RNA probes were validated on human pancreas tissue. In the normal eye, *EPOR *was expressed in the retinal ganglion cell layer. Minimal expression was observed in the inner and outer nuclear layer. Under conditions of diabetic retinopathy, *EPOR *expression shifted to photoreceptor cells. Increased expression was also observed in the peripheral retina.

**Conclusion:**

*EPOR *expression may be a biomarker or contribute to disease mechanisms in diabetic retinopathy.

## Background

Human erythropoietin (EPO) is the primary regulator of erythropoiesis, stimulating growth and promoting differentiation of red blood cell progenitors[1]. The primary stimulus for EPO release is decreased oxygen delivery, most often due to anemia or hypoxia[2]. EPO is an acidic glycoprotein hormone that is produced by the kidney and to a much lesser degree (<10 percent) the liver. EPO binds to transmembrane epogen receptors (EPOR), which are expressed primarily by hematopoietic progenitor cells but also by nonhematopoietic cells and tissues such as endothelial cells, cardiomyocytes, and neurons, the liver, uterus, and retina[3]. EPO also shows angiogenic activity *in vitro *by stimulating vascular endothelial cells to proliferate and migrate[4]. EPO is now also known as a potent antiapoptotic factor for EPOR presenting cells, particularly neural cells[5].

EPO may play a direct role in the pathophysiology of diabetic retinopathy. Vitreous levels of EPO are higher in diabetic patients, suggesting that EPO may be produced as an endogenous neuroprotectant against ischemia. Compared with the proangiogenic vascular endothelial growth factor, EPO is more strongly associated with proliferative diabetic retinopathy than VEGF[6]. In diabetic rats, intravitreal injection of EPO upregulated EPOR in the neurosensory retina and had a protective effect on vascular and photoreceptor cells[7]. In a mouse model of oxygen-induced retinopathy inhibition of EPO by injection of intravitreal EPO siRNA suppressed retinal neovascularization[8]. Inhibition of systemic EPO production has been clinically observed in early diabetic nephropathy and results in anemia that is associated with an aggravated course of DR[9]. Intravenous administration of EPO to treat azotemia-induced anemia in diabetic patients demonstrated a beneficial effect on macular edema and improved visual outcome[10]. In a cross-sectional study of 1691 diabetic patients, the severity of anemia correlated with the severity of PDR[11]. Friedman reported 5 cases in which patients with severe anemia and PDR had substantial reduction of macular hard exudates after treatment with systemic EPO[12].

Identifying the target cells and conditions regulating EPOR expression is important when considering therapeutic intervention. In a study of post-mortem retinas of 9 patients with diabetes but without diabetic retinopathy, EPOR was detected in the neuroretina and in the retinal pigment epithelium. No difference in expression of EPOR between diabetic eyes and non-diabetic was observed eyes[13]. However, they did not report which layers of the neuroretina they detected expression.

Evidence for EPOR localization in mice has been contradictory. Chen et. al showed EPOR to be expressed in all layers of the inner retina and predominantly in the ganglion cell layer[14]. Kilic and associates also showed localization to the ganglion cell layer[15]. However, Grimm and associates have evidence of its localization to photoreceptors[16]. Hypoxia is a potent trigger for EPO and EPOR expression, and a growing body of evidence suggests hypoxia may induce changes in the expression of EPOR in the eye. Compared with age-matched controls, EPO mRNA expression levels are greatly increased in the retinas of mice under hypoxic conditions[14].

While antibodies suitable for EPOR detection in mouse tissues exist, they lack specificity for human EpoR[17]. For this reason mRNA *in situ *hybridization experiments were performed to identify cellular *EPOR *expression in the human diabetic retinopathy eye.

## Results

**Figure 1:**
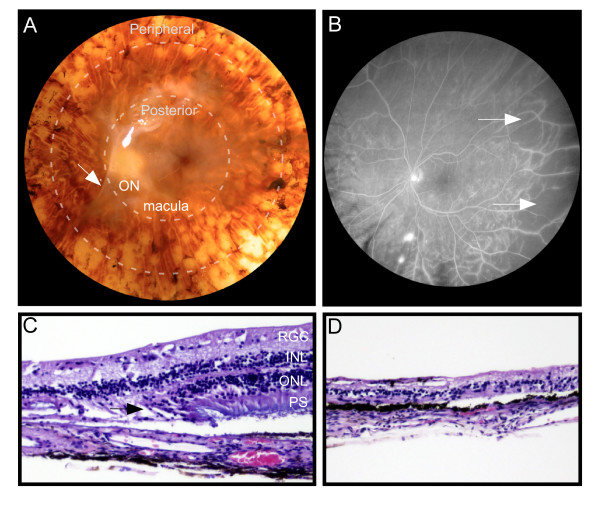
**Regressed Proliferative Diabetic Retinopathy**. **A**, Gross view of eye. There was a membrane (arrow) overlying the macula and optic nerve head (ON). Peripheral to this were laser photocoagulation scars. Dotted lines show the posterior and peripheral retina. **B**, Representative fluorescein angiogram showing capillary dropout (arrows) and peripheral ischemia. **C**, H&E section of the posterior retina shows variable cell and photoreceptor segment loss (arrow). **D**, H&E section of the peripheral retina shows extreme cell loss and disorganized cytoarchitecture. Abbreviations: H&E, heamotoxylin and eosin; ON, optic nerve; RGC, retinal ganglion cell layer; INL, inner nuclear layer; ONL outer nuclear layer; RPE, retinal pigment epithelium layer; PS, photoreceptor segments.

Gross examination of the diabetic eye showed extensive photocoagulation scars throughout the peripheral retina. The vitreous was collapsed and there was a membrane overlying the posterior pole. The macula appeared edematous. (Figure 1A) These findings were consistent with prior treatment of proliferative diabetic retinopathy where the surviving retinal cells would have been subjected to severe ischemia, especially in the peripheral retina. A representative fluorescein angiogram of retinopathy from diabetes is shown. In such cases, there is extensive capillary loss, which is more pronounced in the retinal periphery[18]. (Figure 1B) The eye was sectioned and histological examination of the posterior retina showed preretinal fibrosis, nerve fiber layer edema, variable cell loss especially in the photoreceptor layer, tractional retinal detachment, and retinal pigment epithelial hyperplasia. (Figure 1C) The retina became thinner in the periphery and there was cell loss and abnormal tissue and cytoarchitecture. (Figure 1D)

**Figure 2:**
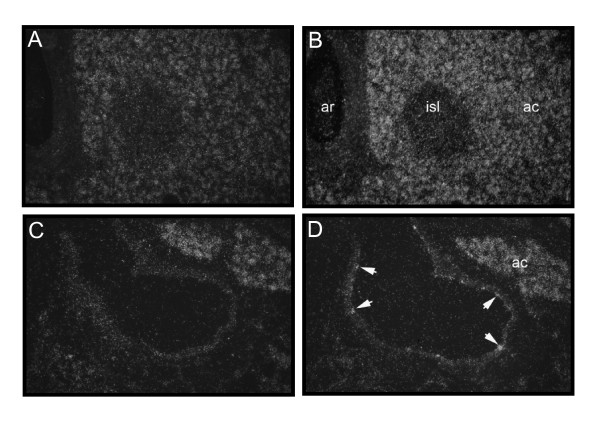
**EpoR probe validation in human pancreatic tissue**. **A**, A section of human pancreatic tissue with sense RNA probes for EpoR. **B**, Same tissue with anti-sense RNA probes for EpoR, demonstrating increased expression in islet of Langerhans cells. **C**, A section of human pancreatic tissue showing a vein adjacent to an acinar cell, with sense RNA probes. **D**, The same tissue with anti-sense RNA probes for EpoR, demonstrating expression in the endothelium of the vein (arrows). Abbreviations: ac, acinar cells; ar, artery; isl, islet of Langerhans.

To better understand *EPOR *expression in the ischemic retina, an *in situ *hybridization assay was developed. To validate the assay, EPOR antisense probes were first applied to human pancreatic sections where *EPOR *is highly expressed. Expression was seen in the acinar cells and vessel lumen endothelial cells as previously described[4,19] (Figure 2). Only a minimal, non-specific signal was observed with the sense probe (Figure 2) or unlabeled probe (data not shown).

**Figure 3:**
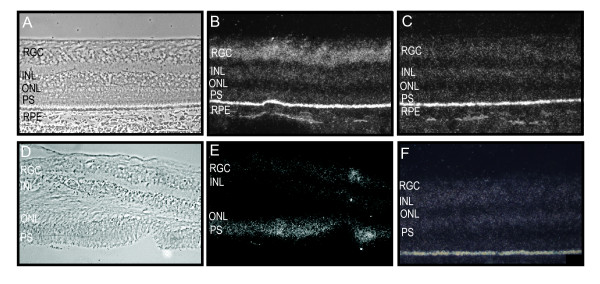
**EpoR mRNA expression in the posterior normal (A-C) and diabetic (D-F) retina**. **A**, Phase contrast micrograph of a normal human retina. **B**, Anti-sense RNA probes for EpoR in normal retina. **C**, Control sense RNA probes for EpoR in normal retina demonstrates expression in the retinal ganglion cell layer. **D**, Phase contrast micrograph of a diabetic human retina with an artifactual detachment. **E**, Anti-sense sense RNA probes for EpoR in diabetic retina. **F**, Control sense RNA probes for EpoR shows decreased expression in the RGC layer and an increase in the photoreceptor segments. Abbreviations: RGC, retinal ganglion cell layer; INL, inner nuclear layer; ONL outer nuclear layer; RPE, retinal pigment epithelium layer; PS, photoreceptor segments.

The posterior retina was then examined. In the normal human eye, *EPOR *was expressed in the retinal ganglion cell layer. No expression was observed in the inner or outer nuclear or plexiform layers. (Figures 3A - C) In contrast, the diabetic retina showed increased expression in the photoreceptor cells in addition to expression in the retinal ganglion cell layer. (Figures 3D - F)

**Figure 4:**
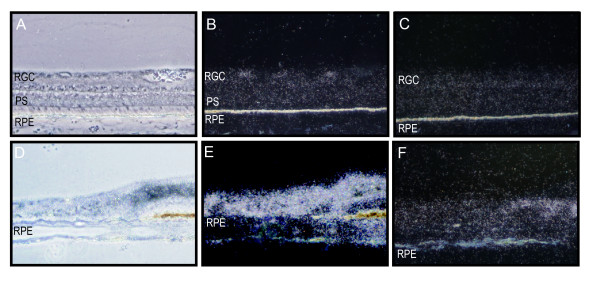
**EpoR mRNA expression in the peripheral normal (A-C) and diabetic (D-F) retina**. **A**, Phase contrast micrograph of the peripheral normal human retina. **B**, Anti-sense RNA probes for EpoR in normal retina demonstrates expression in the few retinal ganglion cells present in the peripheral retina. **C**, Control sense RNA probes for EpoR in normal retina. **D**, Phase contrast micrograph of a diabetic human retina shows loss of cytoarchitecture. **E**, Anti-sense RNA probes for EpoR shows intense signal in the peripheral retina. **F**, Control sense RNA probes for EpoR in diabetic retina. Abbreviations: RGC, retinal ganglion cell layer; INL, inner nuclear layer; ONL outer nuclear layer; RPE, retinal pigment epithelium layer; PS, photoreceptor segments.

In the retinal periphery, the retinal thickness and number of cells normally decreases. In the normal eye there was a corresponding decrease in *EPOR *signal. (Figures 4A - C) In the diabetic retina, however, there was a significantly higher increase in *EPOR *signal. This did not correspond to increased numbers of cells or thicker tissue. Rather, there was loss of cells and disruption of the normal tissue and cytoarchitecture, and it was not possible to assign this expression to a specific cell type or layer (Figures 4D-F). Comparison to control sense probe sections suggested the hybridization signal was not due to increased background.

## Discussion

Reports of expression of EPOR in the human diabetic retina in diabetics is limited without indication of cellular origin. One report detected EPOR in human epiretinal membrane of proliferative diabetic retinopathy[20]. EPOR was detected in retinal and RPE extracts of patients with diabetes, but these patients had no evidence of ischemia or retinopathy[13].

Our results reveal EPOR mRNA is expressed primarily in the ganglion cell layer of the human retina under normal conditions[14,15]. Although absent from in human photoreceptors under normal conditions, our results suggest that EPOR is upregulated in photoreceptors during diabetic retinopathy. The high metabolic rate of dark-adapted photoreceptors can lead to borderline hypoxic levels in the normal retina, so photoreceptors may be particularly susceptible to hypoxia[21]. Our finding of increased EPOR expression in the peripheral retina most likely indicates increased hypoxia/ischemia in these areas. This is consistent with current understanding of the pathogenesis of diabetic retinopathy, in which retinal capillary non-perfusion results in retinal ischemia initially in the mid-retinal periphery[22]. The distortion of the cytoarchitecture of the human diabetic retina in our study is likely a consequence of extensive panretinal photocoagulation treatment. Increased EPOR expression may reflect increased hypoxia, and may be an endogenous attempt by the body to protect the retina from hypoxia with the neuroprotective properties of EPO.

Hypoxia is a potent stimulus of increased EPO production. Retinal EPO mRNA levels were increased in mice in a dose-dependent manner following hypoxia[16]. It is thought that EPO may function as an endogenous neuroprotectant. In a mouse-model of retinal detachment, in which photoreceptors die from ischemia, Xie and colleagues demonstrated that there is upregulation of the EPO-EPOR system[23]. Other studies demonstrated in a mouse-model that levels of both EPO mRNA and EPOR mRNA increased in the retinas of mice during hypoxia-induced retinal neovascularization[8]. The angiogenic properties might be relevant in the peripheral retina, which is the site of neovascularization in proliferative diabetic retinopathy. Increased expression is found in other organ systems, including the spleen and brain, where the EPO/EPOR signaling system is upregulated under conditions of hypoxia[24,25].

The EPO/EPOR signaling system may contribute to the survival of neurons through a variety of mechanisms including inhibition of apoptosis, a reduction in reactive oxidative species, a reduction in proinflammatory cytokines, recruitment of stem cells and maintenance of vascular autoregulation lending protection from ischemic damage[26]. Transcriptional regulation of EPO expression by hypoxia-inducible factor-1 (HIF1α) maintains survival of cone photoreceptors against genetic insults[27]. EPO promotes neural outgrowth from retinal ganglion cells in a dose-dependent manner and preserves their survival after axotomy[28]. Additionally, hypoxia-induced retinal EPO expression appears to protect retinal neurons from transient global ischemic and reperfusion injury through an anti-apoptotic pathway[29]. There is evidence that systemic EPO administration may protect retinal photoreceptors from light-induced apoptotic pathways in retinal degeneration models.

Pathologic angiogenesis is a final common pathway in ischemic ocular diseases. In proliferative diabetic retinopathy, catastrophic vision loss is often the result of neovascular membranes that lead to hemorrhage, fibrosis, and retinal detachment[30]. The destruction of retinal tissue with laser photocoagulation is thought to mitigate retinal ischemia, and remains the established treatment for diabetic retinal neovascularization[30]. The molecular mechanisms underlying the ischemic drive for proliferative diabetic retinopathy are poorly understood, and development of more effective and less destructive therapy is necessary.

## Conclusion

Our findings suggest in the human retina, EPOR mRNA is primarily expressed in the ganglion cell layer. Under conditions of ischemia such as diabetic retinopathy, there may be up-regulation of EPOR expression in the photoreceptors and in the peripheral retina.

Future studies with additional samples may lead to more conclusive answers regarding the potential role of EPO in diabetic retinopathy. Nevertheless, our results support the concept that a dynamic EPO-EPOR signaling system is present in the ischemic retina and may offer a new therapeutic modality for ischemic ophthalmic diseases. Given its neurotrophic properties, EPO may be an ideal candidate to signal retinal ganglion cells or photoreceptors in anterior ischemic optic neuropathy or central retinal artery occlusion where there are currently no effective treatments. Careful inhibition of EPO may prove to be an effective way to treat or prevent diabetic retinopathy and other forms of angiogenesis. Ultimately, clinical application and regulation of the EPO/EPOR system will require careful dosing so that vessel proliferation is inhibited without impairment of neuronal survival.

## Methods

This study adhered to the tenets of the Declaration of Helsinki and was approved by the Institutional Review Board. Post-mortem retinas from two eyes were obtained from a 68 year-old male with regressed proliferative diabetic retinopathy and end-stage renal disease and peripheral vascular disease. This was compared to two retinas form an age and sex-matched donor eyes without diabetes or other ophthalmic pathology. Human retina and archived human pancreatic sections were fixed overnight, dehydrated and infiltrated with paraffin. Serial 5 to 8 μm sections were mounted on gelatin-coated slides, deparaffinized in xylene and rehydrated in a series of ethanols and PBS. The sections were digested with proteinase K, treated with triethanolamine/acetic anhydride, washed and dehydrated.

The cRNA transcripts were synthesized in vitro according to manufacturer's conditions (Ambion) and labeled with ^35^S-UTP (> 1000 Ci/mmol; Amersham). Sections were hybridized overnight at 55°C in 50% deionized formamide, 0.3 M NaCl, 20 mM Tris-HCl pH 7.4, 5 mM EDTA, 10 nM NaPO4, 10% dextran sulphate, 1 × Denhardt's, 50 μg/ml total yeast RNA, and 50-80,000 cpm/μl ^35^S-labeled cRNA probe. The tissue was subjected to stringent washing at 65°C in 50% formamide, 2 × SSC, 10 mM DTT and washed in PBS before treatment with 20 μg/ml RNAse A at 37°C for 30 minutes. Following washes in 2 × SSC and 0.1 × SSC for 10 minutes at 37°C, slides were dehydrated, exposed to x-ray film for 5 days, then dipped in Kodak NTB nuclear track emulsion and exposed for 18 days in light-tight boxes with desiccant at 4°C. Photographic development was carried out in Kodak D-19. Slides were counterstained lightly with hematoxylin and eosin and analyzed using both bright- and darkfield optics. Sense control cRNA probes (identical to the mRNAs) always gave background levels of hybridization signal.

## Competing interests

The authors declare that they have no competing interests.

## Authors' contributions

SST and VBM designed and conducted the experiments and conceived the initial idea. All authors contributed equally in analyzing and interpreting the data. SSS and VBM drafted the manuscript. All authors read and approved the final manuscript.
